# Nano-contact microscopy of supracrystals

**DOI:** 10.3762/bjnano.6.126

**Published:** 2015-05-29

**Authors:** Adam Sweetman, Nicolas Goubet, Ioannis Lekkas, Marie Paule Pileni, Philip Moriarty

**Affiliations:** 1 The School of Physics and Astronomy, The University of Nottingham, Nottingham, NG7 2RD, U.K.; 2 Sorbonne Universités, UPMC Univ Paris 06, UMR 8233, Monaris, F-75005, Paris, France; 3 CNRS, UMR 8233, Monaris, F-75005, Paris, France; 4 CEA/IRAMIS, CEA Saclay, 91191, Gif-sur-Yvette, France

**Keywords:** dynamic force microscopy, nanoparticle, non-contact atomic force microscopy, point contact imaging, scanning probe microscopy, supracrystal

## Abstract

**Background:** Highly ordered three-dimensional colloidal crystals (supracrystals) comprised of 7.4 nm diameter Au nanocrystals (with a 5% size dispersion) have been imaged and analysed using a combination of scanning tunnelling microscopy and dynamic force microscopy.

**Results:** By exploring the evolution of both the force and tunnel current with respect to tip–sample separation, we arrive at the surprising finding that single nanocrystal resolution is readily obtained in tunnelling microscopy images acquired more than 1 nm into the repulsive (i.e., positive force) regime of the probe–nanocrystal interaction potential. Constant height force microscopy has been used to map tip–sample interactions in this regime, revealing inhomogeneities which arise from the convolution of the tip structure with the ligand distribution at the nanocrystal surface.

**Conclusion:** Our combined STM–AFM measurements show that the contrast mechanism underpinning high resolution imaging of nanoparticle supracrystals involves a form of nanoscale contact imaging, rather than the through-vacuum tunnelling which underpins traditional tunnelling microscopy and spectroscopy.

## Introduction

Artificial solids comprising extended assemblies of nanocrystals with a narrow size distribution represent an especially important class of nanostructured material. In addition to their inherent tunability, this type of “designer” solid is of particular interest in the context of the evolution of mesoscopic and, indeed, macroscopic physical properties from nanoscale components [1–3]. Remarkably well-ordered 3D nanocrystal superlattices, otherwise known as supracrystals [4], can now be synthesized via slow nucleation and growth under a solvent atmosphere [5]. This assembly process produces not only supracrystalline films (which nucleate and grow at the air–solvent interface) but also large polyhedral supracrystals arising from precipitation in the colloidal suspension.

There is a significant body of work that focuses on elucidating the electronic properties of nanocrystals and their associated 1D, 2D, and 3D assemblies. Building on the conceptual and theoretical framework put forward by Middleton and Wingreen in the early 1990s [6], a number of groups [7–11] have shown that the current–voltage (*I*(*V*)) characteristics of nanocrystal superlattices follow a power law dependence above a voltage threshold (which is related to the Coulomb gap for the system). The power law exponent depends both on the effective dimensionality and the amount of topological/charge disorder in the system. The distribution of nanocrystal connectivity due to this disorder plays an essential role in determining the topological “landscape” for charge transport, which can be affected at the nanoscopic, mesoscopic, and microscopic scales [12].

Although a similar power law behaviour has been observed for the *I*(*V*) characteristics of both interfacial and precipitated supracrystals, Yang et al. [11,13] point out that it is somewhat counter-intuitive and surprising that supracrystals that are of order 5 μm thick (i.e, ≈700 nanoparticle layers) are sufficiently conductive for STM and scanning tunnelling spectroscopy (STS) studies. However, not only are STM and STS measurements possible, but the quality of imaging is comparable to that attained on monolayer (or submonolayer) coverages of nanoparticles on various substrates [14–15] (see, in particular, Figure 7a of Yang et al. [11].) However, bias voltages significantly higher than those conventionally used in STM measurements (as high as 9 V [13]) were sometimes necessary to image thick precipitated supracrystals. In addition to the unexpected imaging resolution, tunnelling spectra of nanocrystals were interpreted as exhibiting the collective behaviour of the ensemble with the spectral fingerprint of an isolated nanocrystal superimposed on the overall *I*(*V*) characteristic [13].

Here we extend scanning probe measurements of supracrystals to an analysis based on a combination of STM and dynamic force microscopy (DFM) imaging and spectroscopy. DFM experiments, also known as non-contact AFM (NC-AFM), are carried out using a quartz tuning fork sensor in the qPlus geometry [16–17] to which a tip has been glued. Shifts in the resonant frequency of a tine of the tuning fork due to variations in the tip–sample interaction are tracked and, via the formula introduced by Sader and Jarvis [18], can be converted to force or potential energy measurements. The qPlus sensor facilitates, in principle, a straight-forward method of acquiring tunnelling current and tip–sample force maps in parallel, but there are important instrumental artefacts [19–20] and physical effects [21] that can produce crosstalk between the measurement channels and these need to be carefully considered. With this proviso in mind, the DFM-STM combination can be exploited to correlate, in parallel, the dependence of the tunnel current and the tip–sample force on the displacement of the probe.

Our combined STM-DFM measurements clearly show that STM imaging of low conductivity supracrystals involves a contact conduction mechanism, and not the through-vacuum tunnelling that is exploited in conventional tunnelling microscopy. The possibility of atomic scale point-contact imaging in STM was recognised by Smith et al. almost three decades ago [22], and in the intervening years, the relationship between the variation in the tunnel current and the tip–sample force as a function of probe displacement has been studied in considerable detail [23–27]. A recent review [28] outlines key developments in point-contact measurements, including the quantum point-contact microscopy strategy introduced by Zhang et al. [29]. State-of-the-art qPlus DFM, where both intra- [30–31] and inter-molecular [32–34] resolution are increasingly the norm, also exploits imaging in the point-contact regime for which electron repulsion underpins the contrast mechanism (as pioneered by Gross et al. [30]). We apply a similar type of contact imaging protocol to nanocrystal superlattices for the first time and demonstrate that subparticle resolution images can be acquired in constant height mode, despite the high curvature of the particle surfaces. There remains, of course, the perennial issue plaguing the interpretation of scanning probe microscopy images: the convolution of the tip and sample structure. Nonetheless, our results clearly show that there is significant potential for qPlus DFM imaging to provide high resolution images of the surfaces of nanoparticles and, for example, to lay to rest the controversy regarding molecular self-assembly and phase separation in the ligand shell of nanoparticles [35].

## Experimental

The Au nanocrystals used here were synthesized using a modified organometallic reduction method [36]. Briefly, 0.25 mmol of chloro(triphenylphosphine)gold(I) was dissolved in 25 mL of toluene and 250 μL of dodecanethiol. The reducing solution was made with 2.5 mmol of tertbutylamine borane complex dissolved in 15 mL of toluene. Both solutions were heated to 100 °C and mixed together. The colourless reaction medium first turns to brown and then quickly to dark red. After five minutes the solution was allowed to cool to room temperature. The resulting nanocrystals have a mean diameter of 7.4 nm with 5% polydispersity (see Supporting Information File 1).

A portion of the colloidal solution was washed with ethanol to produce the starting solution for Au nanocrystal superlattices. The resulting precipitate was then redispersed in toluene and the self-assembly process occurred in the washed solution in a toluene-saturated atmosphere. After one week without evaporation, the toluene/air interface exhibited a deposit which appeared golden in colour [4]. A part of this interfacial deposit was withdrawn from the interface solution with a DuNouy ring and deposited on a highly oriented pyrolytic graphite substrate. High-resolution scanning electron microscopy pictures revealed that the nanocrystals organise into compact structures (see Supporting Information File 1). We note that the samples were not annealed or treated in any other way before the scanning probe experiments were undertaken.

The scanning probe data in this paper were acquired using an Omicron Nanotechnology combined low temperature STM/DFM operating under UHV conditions at cryogenic temperatures (78 K, with liquid nitrogen cooling). Commercial qPlus sensors from Omicron with an electrochemically etched tungsten wire glued to one tine of the tuning fork were first prepared on clean Si(111)-(7 × 7) samples by standard STM techniques before imaging of the nanocrystal samples. During imaging of the supracrystal surface, spontaneous and regular tip changes were observed, thus it is possible that the qPlus probe became nanocrystal- (or thiol-)terminated. Imaging was performed in constant current STM, constant frequency shift (Δ*f*) DFM, and constant height DFM modes. In addition to traditional STM, we also carried out dynamic STM (dSTM) imaging and spectroscopy, where the tip was oscillated with a small amplitude (of order 0.1−0.3 nm, see below) normal to the surface. The use of constant height imaging (using a similar protocol to that described previously [30,33]) allows us to probe the tip–sample interaction on the repulsive branch of the frequency shift curve, which is typically not available using conventional Δ*f* feedback on the attractive branch.

Oscillation amplitudes (*A*_0_) between 0.1 and 0.3 nm were typically used for DFM imaging. We reduced any possible electronic crosstalk [19] or so-called “phantom force” [21] effects by ensuring DFM imaging was performed in the absence of a detectable tunnel current. Normally this was done by ensuring the gap voltage was set to 0 V, but we were also able to make force measurements without detectable tunnel currents at higher voltages (see Results and Discussion section). In the experimental set-up used for the experiments described in this paper, the tip was held at ground potential and the sample was biased. To help stabilise the imaging conditions, a custom-built atom tracking system [37] was used to apply feed-forward correction to reduce the effect of thermal drift and piezo-electric creep.

To measure the site-specific force between the probe tip and a single nanocrystal, single-point Δ*f*(*z*) spectroscopy measurements were acquired both on a particle (so-called “on” spectra), and in a region not demonstrating any site-specific interaction (so-called “off” spectra). The non-site-specific interactions were then subtracted from the “on” spectra [23,38] and the resultant short-range Δ*f*(*z*) was inverted to extract force values using the Sader–Jarvis algorithm [18,39].

## Results and Discussion

**Figure 1:**
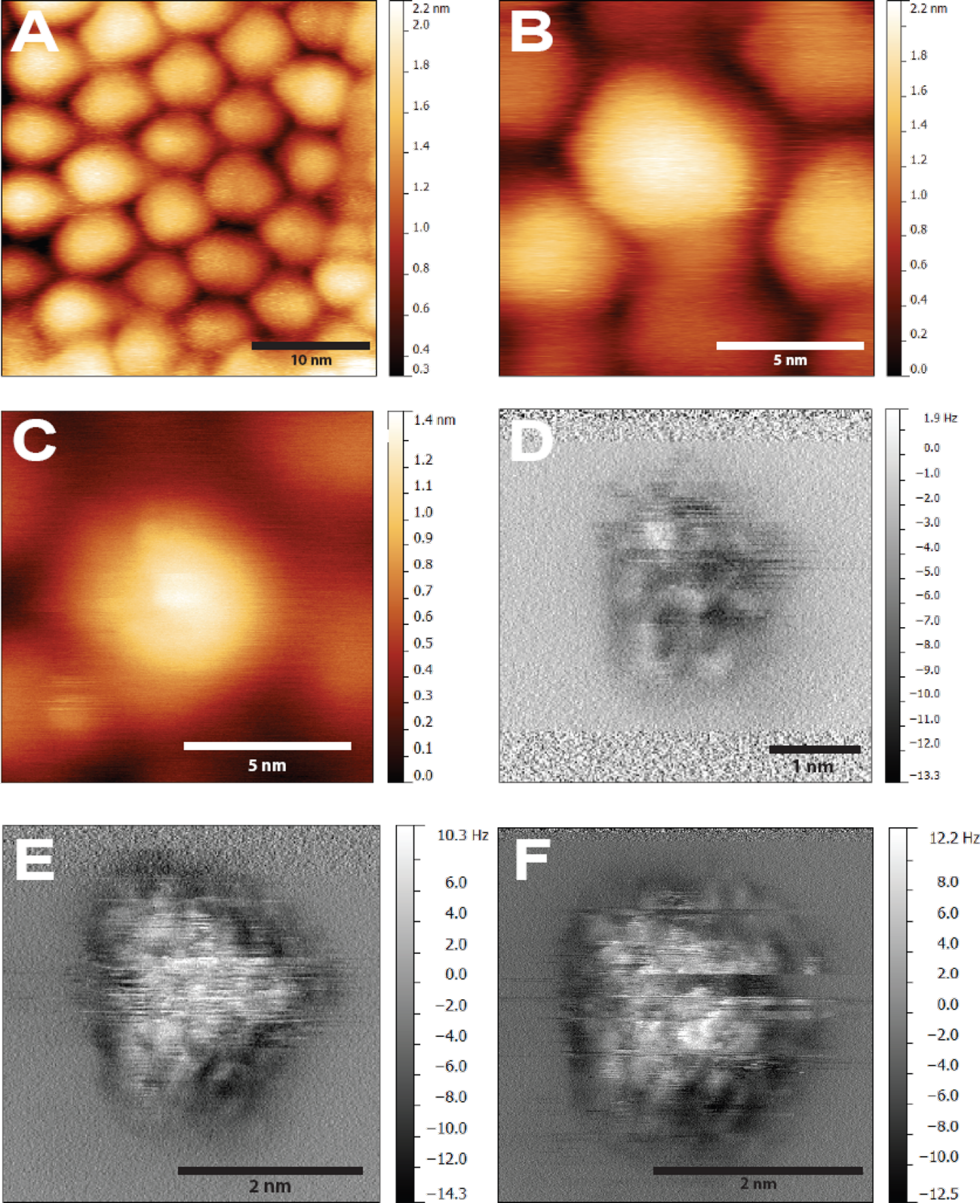
(A) Overview dSTM image showing packing of nanocrystals in a supracrystal. *V*_gap_ = +2.5 V, 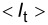 = 20 pA, *A*_0_ = 1 nm. (B) High resolution dSTM image of nanocrystals. *V*_gap_ = +1.5 V, 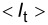 = 10 pA, *A*_0_ = 0.11 nm. (C) Constant frequency shift DFM image of same region *V*_gap_ = 0 V, Δ*f* = −2 Hz, *A*_0_ = 0.11 nm. (D) Constant height image of nearby region. Estimated height relative to feedback position Δ*z* = −0.2 nm. (E) Constant height DFM, Δ*z* = −0.25 nm. (F) Δ*z* = −0.3 nm. The frequent discontinuities (“slicing”) in E and F are due to modifications/relaxation of the tip–sample junction. We also note that, due to instrumental drift and creep, the Δ*z* values are likely to be systematically underestimated.

In agreement with previous studies [11,13] we readily (within minutes) obtained dSTM images of the supracrystals upon approaching the tip to the sample. Figure 1A,B show constant current dSTM images obtained at moderate tip–sample biases (+2.5 V and +1.5 V respectively). After ensuring the imaging and scan conditions were stable, the tip was retracted several nm and the bias was slowly reduced to 0 V. We then reapproached the sample in constant Δ*f* feedback and slowly increased the Δ*f* setpoint until stable, high contrast DFM imaging was obtained. Figure 1C is an image of the same nanocrystal as shown in the centre of Figure 1B acquired in constant Δ*f* DFM mode at a Δ*f* of −2 Hz. We note that the appearance of the nanocrystals in DFM feedback is broadly comparable to that in dSTM, with the particles having the same approximate size and shape with little internal contrast.

After completing the DFM scan, the tip was positioned over the centre of a nanocrystal and the feedback loop turned off. The same region was then imaged in constant height DFM mode, producing a map of Δ*f*, with dark regions corresponding to attractive interaction (more negative frequency shift), and brighter regions corresponding to repulsive interactions (more positive frequency shifts). The result of imaging the nanocrystal at progressively smaller tip–sample separations is shown in Figure 1D–F.

Due to the nature of operation, we only observe the very top of the nanocrystal in constant height mode , with the surrounding regions imaging as a featureless void. Nonetheless, a number of additional features become apparent in constant height imaging. The crystalline shape of the nanocrystal is apparently clearer in constant height mode, with a triangular appearance suggestive of faceting, as proposed recently by Goubet et al. [40]. In addition, we see an internal structure that is not apparent in either the dSTM or constant Δ*f* DFM topographs. Interpreting these intra-nanocrystal features is challenging for a number of reasons. First, the exact nature of our tip apex is unknown, and very possibly terminated either by free thiol ligands or entire nanocrystals. Second, due to the high aspect ratio of the nanocrystal surface (as compared to an atomically flat substrate), it is difficult to image a very large area of a particle without causing very close approach over the topmost regions. Consequently, we often see a number of “slices” and tip changes during imaging, suggesting that either the apex of the tip or the coating of the nanocrystal is undergoing changes during scanning. As such, we cannot assume a priori that a simple single atom “point-like” contact is responsible for the observed contrast. Finally, the supracrystal is composed of a mixture of single domain and polycrystalline nanocrystals [40], complicating the analysis of the DFM images.

**Figure 2:**
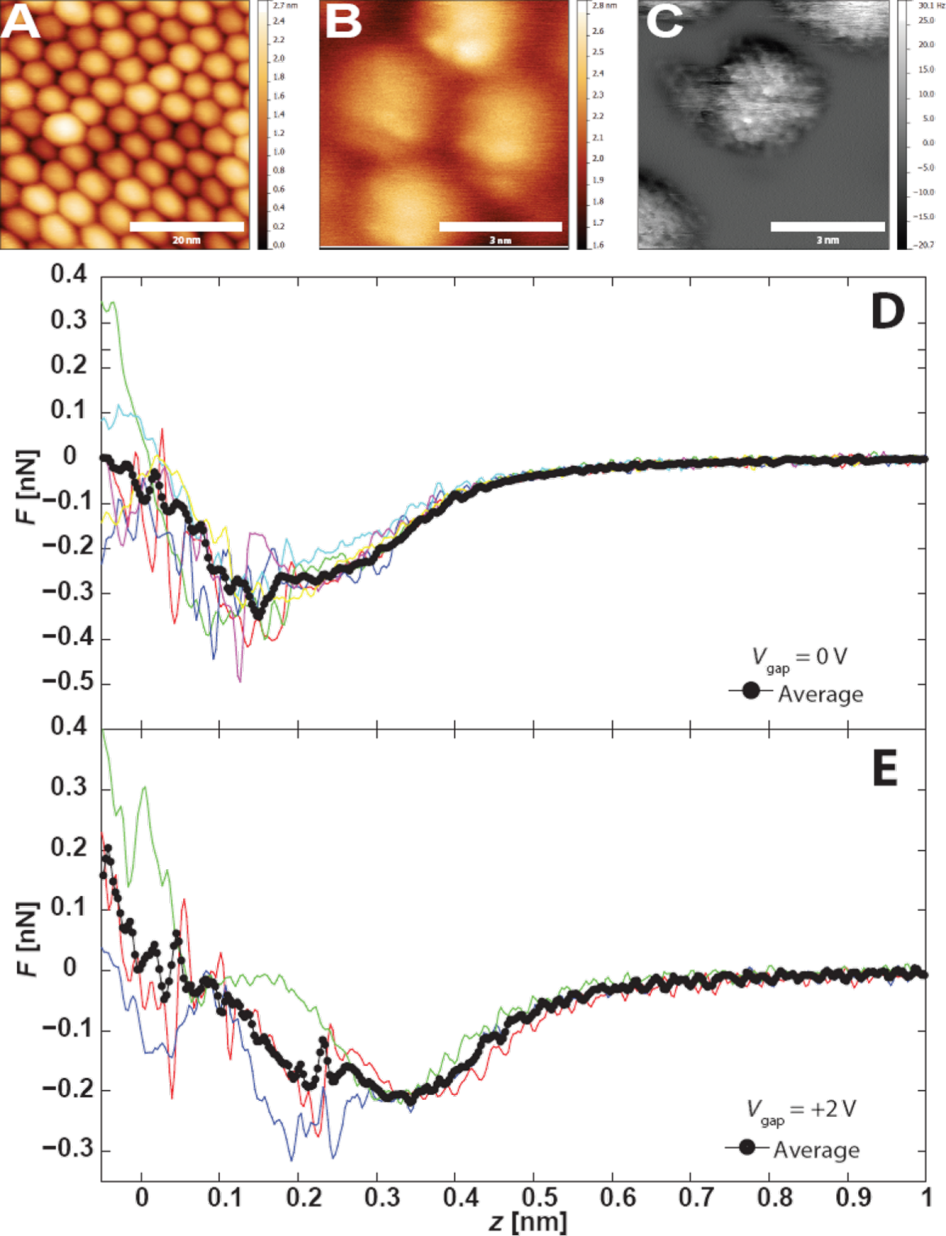
(A) Overview dSTM showing nanocrystal assembly, *V*_gap_ = +2 V, 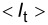 = 20 pA. (B) DFM image acquired in the region shown in A. *V*_gap_ = 0 V, Δ*f* = −2.3 Hz, *A*_0_ = 0.11 nm. (C) Constant height Δ*f* image of nearby region Δ*z* = −0.7 nm. (D) Site-specific tip–sample force data taken over a single nanocrystal with zero applied bias. (E) Site-specific tip–sample force data taken over the same nanocrystal and height range with an applied bias of +2 V. Note that no tunnel current was detected during this measurement.

We found that the evolution in contrast from STM, to constant Δ*f* DFM, to constant height DFM was reproducible if we used different tip apices (prepared via gentle crashing into the sample). Figure 2A–C shows a dSTM, constant Δ*f* DFM, and constant height DFM image all acquired using a different tip apex over a different region of the sample. During this imaging sequence we also measured the quantitative force data shown in Figure 2D,E. The peak attractive force is of order 200−400 pN, comparable to that measured between two weakly interacting (C_60_) molecules, where the interaction is entirely due to dispersion forces [41]. However, while force–distance measurements of single molecule interactions using this technique are typically highly reproducible [31,33,41–43], we observe a very large degree of variation between different *F*(*z*) spectra for the nanocrystals. Although the broad trends remain similar, there are multiple jumps and discontinuities. A plausible explanation is that while the macroscopic apex of the tip remains stable, the very apex of the tip is undergoing multiple reconfiguration events upon contact with the sample. This is perhaps unsurprising if we consider the quite likely scenario where we have both a thiol-terminated tip and thiol-coated nanocrystal, and where the thiols on both the tip and nanocrystal would be relatively free to relax as the tip approaches the sample. These observations are also consistent with the numerous reconfiguration events that we observe during constant height imaging (observed, for example, as discontinuities in Figure 1F).

An important conclusion from Figure 2D,E is that the constant height imaging we perform is clearly within the repulsive branch of the short range force, and consequently the tip is in contact with the sample. Despite the strong forces applied to the sample, the supracrystal remains stable, most likely due to the high cohesive energy arising both from the integrated van der Waals forces and ligand interdigitation. The question of where to define the contact point is, of course, a notoriously vexed issue, as Smith et al. pointed out in their pioneering paper on point-contact microscopy using STM [22]. However, regardless of whether we define the contact point as the tip–sample separation associated with the minimum of the potential energy curve (i.e., where *F*(*z*) = 0) or as the point at which the gradient of the force curve changes from positive to negative, it is clear that the STM images are acquired far beyond the tunnelling regime, in a point-contact mode.

In order to investigate the force/tunnel current relationship for this system in more detail, we gradually increased the applied bias from 0 V to +2 V, maintaining the same tip–sample separation. Repeating the force–distance measurements (Figure 2E), we observed a slight modification to the tip–sample force profiles, although we cannot rule out that the reduced peak force could also arise from a minor change in the tip apex. More striking was the observation that even at +2 V bias we observed no detectable tunnel current signal throughout the spectroscopy or imaging, despite clearly being in contact with the sample.

**Figure 3:**
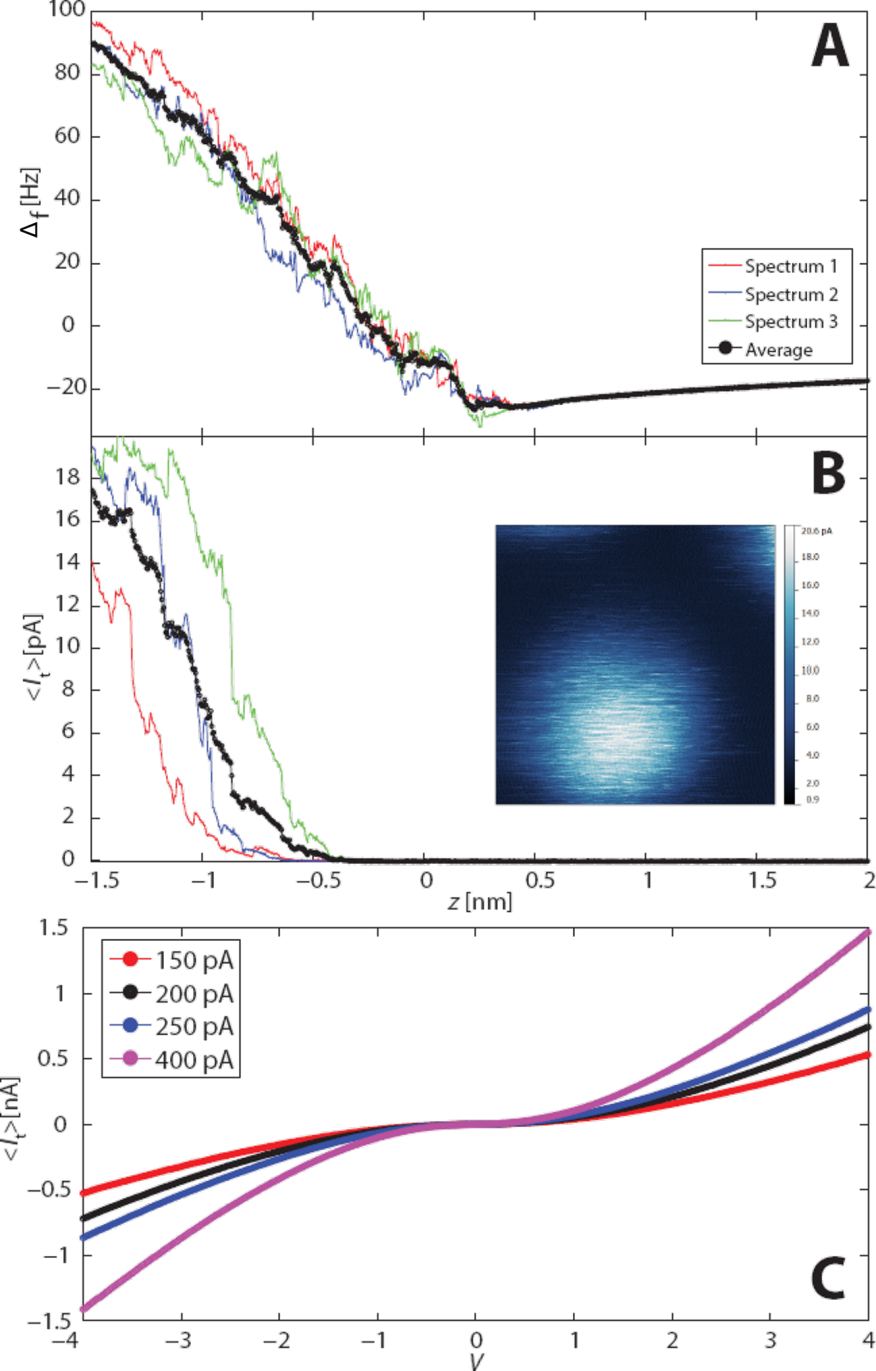
Simultaneous Δ*f* (A) and *I*_t_ (B) data acquired over a single nanocrystal with an applied bias of *V*_gap_ = +2 V. Note the absolute *z* positioning is identical to that used in Figure 2. Inset: Tunnel current map for an individual nanocrystal acquired immediately after the constant height DFM images shown in Figure 2. (C) *I*–*V* data acquired using a different tip apex at various feedback stabilisation currents (stabilisation bias +2 V throughout).

Having previously successfully acquired STM images in the same region at the same bias, we performed combined Δ*f*(*z*) and *I*(*z*) measurements with increasing tip indentation in the same region (Figure 3A). We only began to detect comparable tunnel current signals to the STM setpoint at indentations of −1 to −1.5 nm closer to the sample than the constant height imaging position (Figure 3B), which we previously established was already at a tip–sample separation corresponding to the repulsive branch of the short range force curve. The simultaneously acquired Δ*f* curve also shows strongly repulsive behaviour, but we note that the quantitative short range force cannot be extracted in this case as at this level of indentation there is no complementary “off” curve position that does not show site-specific interactions. Nonetheless, the Δ*f* behaviour provides strong complementary evidence for a repulsive interaction at the tip height at which a detectable tunnel current is observed. Likewise, decreasing the tip–sample separation whilst imaging in constant height mode over the same region provides additional supporting evidence (inset to Figure 3B), showing a comparable tunnel current image to the previous STM imaging at a tip height −0.5 nm closer to the sample than the constant height DFM imaging shown in Figure 2C.

We note that the *I*(*V*) characteristics of the supracrystals (Figure 3C) are broadly in line with those measured by Yang et al. [11] in that they are symmetric about 0 V. A key difference is that we do not observe a Coulomb gap (nor Coulomb staircase) due to the higher temperature of the sample in our experiment (77 K, as compared to Yang et al.’s measurements at 5 K.) In addition, we plot the average tunnel current values acquired over the oscillation cycle of the tip in Figure 3C, rather than the value of the current for a static tip. Nonetheless, there is a clear, almost linear dependence of the tip–sample resistance at the maximum voltage applied (4 V) on the setpoint “stabilisation” current (*I*_s_) used to acquire each of the spectra observed in Figure 3C. The stabilisation current is the setpoint value at which the feedback loop operates before the loop is disabled to allow the *I*(*V*) measurement to take place. *I*_s_ therefore provides a measure of the tip–sample separation in traditional STM where a tunnel gap exists. In our case, the stabilisation current is most likely related to the degree to which the tip is indented into the nanocrystal sample.

## Conclusion

In conclusion, we have presented the first combined STM/DFM study of nanocrystal supracrystals. We readily obtain single nanocrystal resolution in STM, but are only able to resolve subparticle features by operating in constant height DFM mode. The examination of quantitative short range force spectra reveals that STM imaging occurs not by vacuum tunnelling, but by contact imaging in the repulsive force regime.

## Supporting Information

File 1 Size distribution of Au nanocrystals used in our study and scanning electron microscope images of an interfacial supracrystal.
