# Supplementary material for: Nano-contact microscopy of supracrystals
Source: Beilstein J Nanotechnol. 2015 May 29;6:1229–36. doi: 10.3762/bjnano.6.126 (PMC4462851; doi:10.3762/bjnano.6.126)
Supplement: File 1 — Size distribution of Au nanocrystals used in our study and scanning electron microscope images of an interfacial supracrystal. [file Beilstein_J_Nanotechnol-06-1229-s001.pdf]

Supporting Information

for

# Nano-contact microscopy of supracrystals

Adam Sweetman[*1], Nicolas Goubet[2,3], Ioannis Lekkas[1], Marie Paule Pileni[2,3,4] and Philip Moriarty[1]

Address: [1]The School of Physics and Astronomy, The University of Nottingham, Nottingham, NG7 2RD, U.K.; [2]Sorbonne Universités, UPMC Univ Paris 06, UMR 8233, Monaris, F-75005, Paris, France; [3]CNRS, UMR 8233, Monaris, F-75005, Paris, France and [4]CEA/IRAMIS, CEA Saclay, 91191, Gif-sur-Yvette, France

Email: Adam Sweetman - adam.sweetman@nottingham.ac.uk
[*] Corresponding author

# Additional Experimental Data

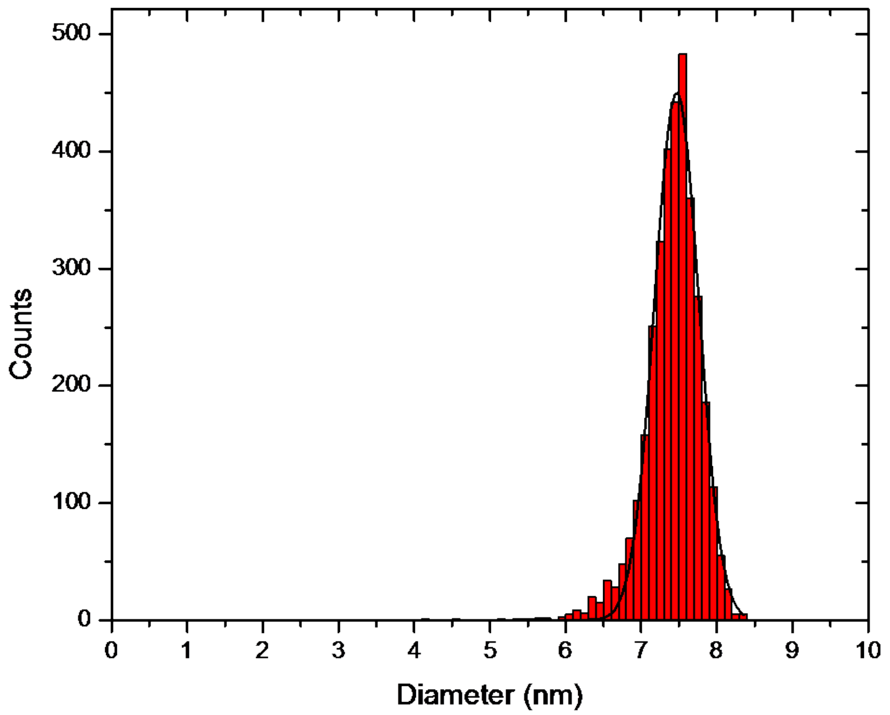


**Figure S1:** Size distribution of the gold nanocrystals used in our study. The dark curve represents a Gaussian fit with a mean diameter of 7.4 nm and polydispersity of 5%.

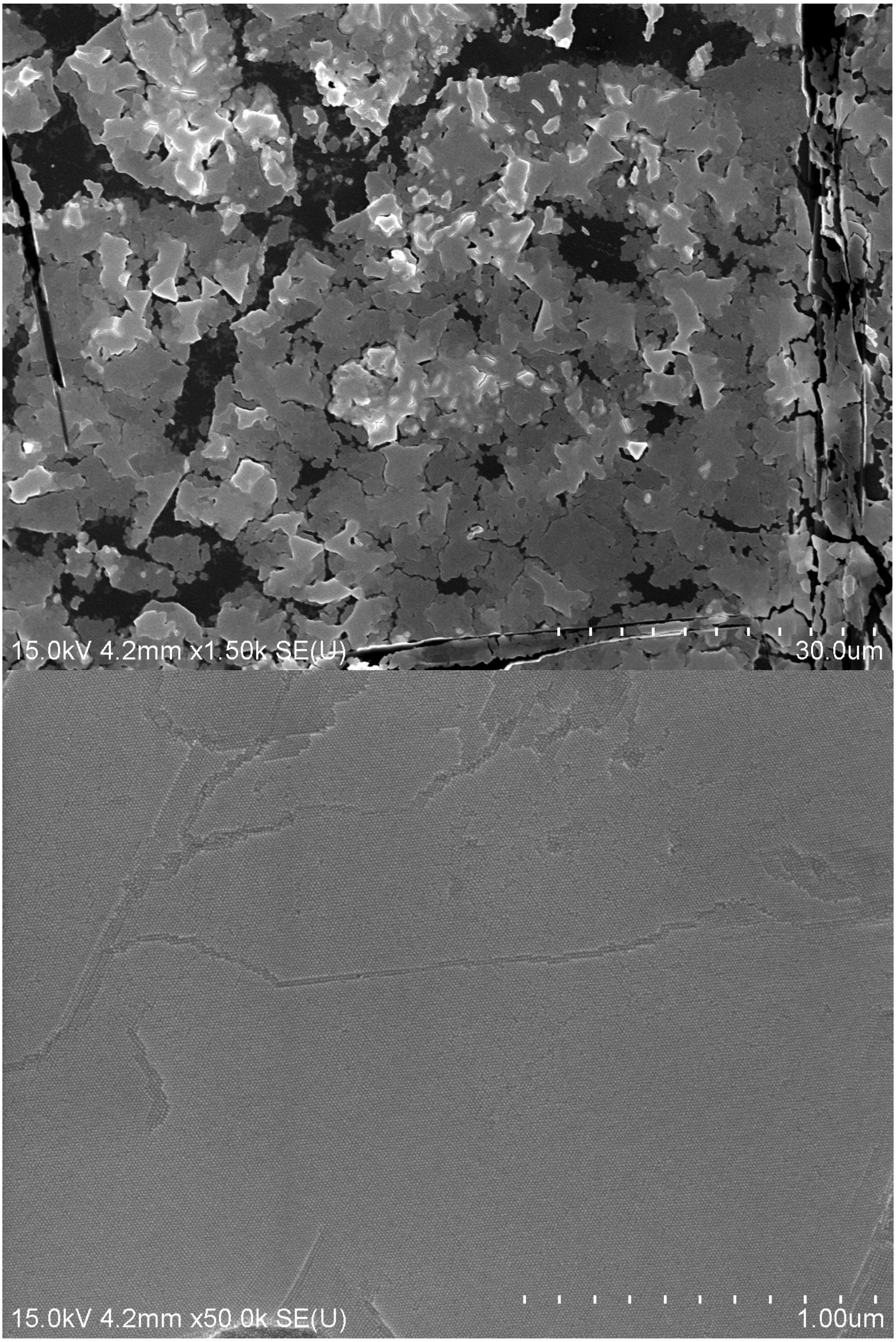


**Figure S2:** Low and high resolution scanning electron microscope images of the interfacial supracrystal made of gold nanocrystals.
